# Role of oxidative stress in the concurrent development of osteoporosis and tendinopathy: Emerging challenges and prospects for treatment modalities

**DOI:** 10.1111/jcmm.18508

**Published:** 2024-07-02

**Authors:** Xianting Xia, Zhengyuan Fang, Yinhua Qian, Yu Zhou, Haoqiang Huang, Feng Xu, Zhiwen Luo, Qing Wang

**Affiliations:** ^1^ Department of Orthopaedics Kunshan Sixth People's Hospital Kunshan Jiangsu China; ^2^ The First Affiliated Hospital of Dalian Medical University Dalian Medical University Dalian Liaoning China; ^3^ Department of Orthopaedics Kunshan Hospital of Chinese Medicine Kunshan Jiangsu China; ^4^ Department of Sports Medicine Huashan Hospital, Fudan Universtiy Shanghai China

**Keywords:** antioxidants, comorbidity, osteoporosis, oxidative stress, reactive oxygen species (ROS), tendinopathy

## Abstract

Both osteoporosis and tendinopathy are widely prevalent disorders, encountered in diverse medical contexts. Whilst each condition has distinct pathophysiological characteristics, they share several risk factors and underlying causes. Notably, oxidative stress emerges as a crucial intersecting factor, playing a pivotal role in the onset and progression of both diseases. This imbalance arises from a dysregulation in generating and neutralising reactive oxygen species (ROS), leading to an abnormal oxidative environment. Elevated levels of ROS can induce multiple cellular disruptions, such as cytotoxicity, apoptosis activation and reduced cell function, contributing to tissue deterioration and weakening the structural integrity of bones and tendons. Antioxidants are substances that can prevent or slow down the oxidation process, including Vitamin C, melatonin, resveratrol, anthocyanins and so on, demonstrating potential in treating these overlapping disorders. This comprehensive review aims to elucidate the complex role of oxidative stress within the interlinked pathways of these comorbid conditions. By integrating contemporary research and empirical findings, our objective is to outline new conceptual models and innovative treatment strategies for effectively managing these prevalent diseases. This review underscores the importance of further in‐depth research to validate the efficacy of antioxidants and traditional Chinese medicine in treatment plans, as well as to explore targeted interventions focused on oxidative stress as promising areas for future medical advancements.

## 1 INTRODUCTION

Osteoporosis, a prevalent metabolic disorder affecting bones, represents a significant global health concern. This condition primarily deteriorates bone microarchitecture and decreases bone mineral density, leading to increased skeletal fragility.^1^, ^2^ As a result, individuals suffering from osteoporosis face increased risks of fractures, which can lead to severe complications such as pain, skeletal deformities and other related morbidities.^1^, ^3^, ^4^ The ageing population in China is contributing to the rising prevalence of osteoporosis, significantly impacting the health and quality of life of the elderly.^1^, ^5^ Osteoporosis has extensive consequences; it often leads to a reduced ability for self‐care following fractures, demanding substantial financial and caregiving resources, thus exacerbating the burden on healthcare systems.^2^, ^6^ Current treatments for osteoporosis, including bone resorption inhibitors, agents promoting bone formation, and mineral supplements such as calcitonin, bisphosphonates and fluorides, mainly provide symptomatic relief.^7^, ^8^ They help to alleviate symptoms and slow the progression of the disease but are often inadequate in restoring the balance between bone formation and resorption.^1^, ^9^ Additionally, these treatments can cause adverse effects and are often expensive. Hence, developing safe and effective prevention and treatment strategies for osteoporosis is critically important.^10^

Chronic tendinopathy, a prevalent musculoskeletal disorder, is characterized by persistent pain, swelling and functional impairment in tendons and nearby tissues.^11^, ^12^ This condition often results in prolonged or irreversible functional limitations. It particularly affects groups such as professional athletes and the elderly.^13^, ^14^ Older individuals, especially those above 70, have a significantly higher incidence of rotator cuff tears compared to younger age groups, with the prevalence increasing dramatically in people over 80.^15^, ^16^ The regenerative capacity of tendon tissues is inherently constrained. These tissues are predominantly composed of collagen and have limited cellularity and blood supply.^17^, ^18^ Early‐stage tendinopathy is usually managed with conservative treatments like medication, mechanical loading and shockwave therapy, whereas more advanced cases may require surgical intervention.^19^, ^20^ However, conservative treatments often provide only temporary relief, and surgery carries risks like infection, joint stiffness, and the possibility of tendon re‐tearing, significantly impacting the patient's quality of life.^21^, ^22^ The high incidence of tendinopathy, its stubborn nature, and the risk of permanent functional damage contribute to a substantial economic burden globally.^23^, ^24^ Current treatment options are limited and frequently result in dissatisfactory clinical outcomes, leading to an increasing interest in researching more effective molecular treatments based on the pathophysiology of tendinopathy.^25^, ^26^

Osteoporosis and chronic tendinopathy are frequently observed in middle‐aged and elderly populations and commonly co‐exist in clinical observations.^27^, ^28^ These conditions share risk factors such as ageing, smoking, high cholesterol, diabetes and genetic predispositions, suggesting potential common pathological mechanisms, including inflammation, immune dysregulation, and oxidative stress.^29^, ^30^ Inflammation, a natural response to harmful stimuli, has been linked to tendinopathy through the involvement of inflammatory cells and mediators in suboptimal tendon healing.^31^, ^32^ Similarly, chronic inflammation often leads to bone loss.^33^, ^34^ Factors such as decreasing oestrogen levels and ageing contribute to systemic inflammation, thereby exacerbating bone resorption, and leading to osteoporosis.^35^, ^36^ The immune microenvironment plays a significant role in the development and progression of osteoporosis, with immune cells and cytokines influencing bone cell functions through pathways like RANK/RANKL/OPG, resulting in an imbalance in bone homeostasis.^37^, ^38^ These cytokines and mediators affect the proliferation and activity of bone cells. They are involved in bone remodelling processes.^39^ Age‐related skeletal and muscle degeneration can be accelerated by impaired immune responses and chronic low‐grade inflammation.^40^, ^41^ In osteoporosis treatment, managing the body's immune response is essential for therapeutic success.^42^ A study by Chen et al. highlighted that hBMSC‐CM can influence macrophage polarisation in vivo and in vitro through the Smad2/3 pathway, thereby aiding tendon‐bone healing due to its immunomodulatory effects.^32^

In recent years, an increasing number of studies have highlighted the significant role of oxidative stress in the pathogenesis of both osteoporosis and tendinopathy.^31^ Oxidative stress refers to the imbalance between the generation and elimination of reactive oxygen species (ROS). Excessive levels induce cellular damage and apoptosis, impacting cellular functions and triggering diseases.^32^ Factors causing oxidative stress include diet, lifestyle, environmental factors, and self‐immune responses.^33^, ^34^ Long‐term oxidative stress can result in chronic inflammation and neurodegeneration, among other detrimental effects.^35^ ROS plays a pivotal role in a variety of diseases. For instance, ROS can directly oxidize and damage pancreatic β‐cells, induce β‐cell apoptosis, and indirectly inhibit β‐cell function by affecting insulin signalling pathways, thereby causing fluctuations in blood sugar levels.^36^, ^37^ ROS also act as functional molecular signals to activate various stress‐sensitive signalling pathways, resulting in insulin resistance. In atherosclerosis, ROS can oxidize LDL into ox‐LDL, which stimulates endothelial cells to secrete multiple inflammatory factors, induces monocyte adhesion and migration into the arterial intima, and inhibits nitric oxide production and its biological activity, thereby accelerating the progression of atherosclerosis.^34^, ^38^ Moreover, oxidative stress is closely related to hypertension, cancer, Alzheimer's disease, arthritis, and Parkinson's disease, among others.^32^, ^39^, ^40^, ^41^, ^42^, ^43^, ^44^ Research has found that oxidative stress plays a vital role in the pathophysiological mechanisms of osteoporosis and tendinopathy.^45^, ^46^ This review aims to provide new insights into the combined treatment of osteoporosis and tendinopathy by elucidating the role of oxidative stress in their shared pathogenic mechanisms.

## 2 OXIDATIVE STRESS AND TENDINOPATHY

Oxidative stress plays a pivotal role in initiating tissue degeneration and damage. During physical activity, tendons continually generate ROS. When ROS production exceeds the tendon's antioxidative capacity, excessive ROS may trigger inflammatory responses and ultimately lead to tendon degeneration and injury.^45^, ^46^ Tendon tissues inherently lack sufficient blood supply, exacerbating hypoxia and increasing vulnerability to oxidative stress during tissue damage.

**FIGURE 1:**
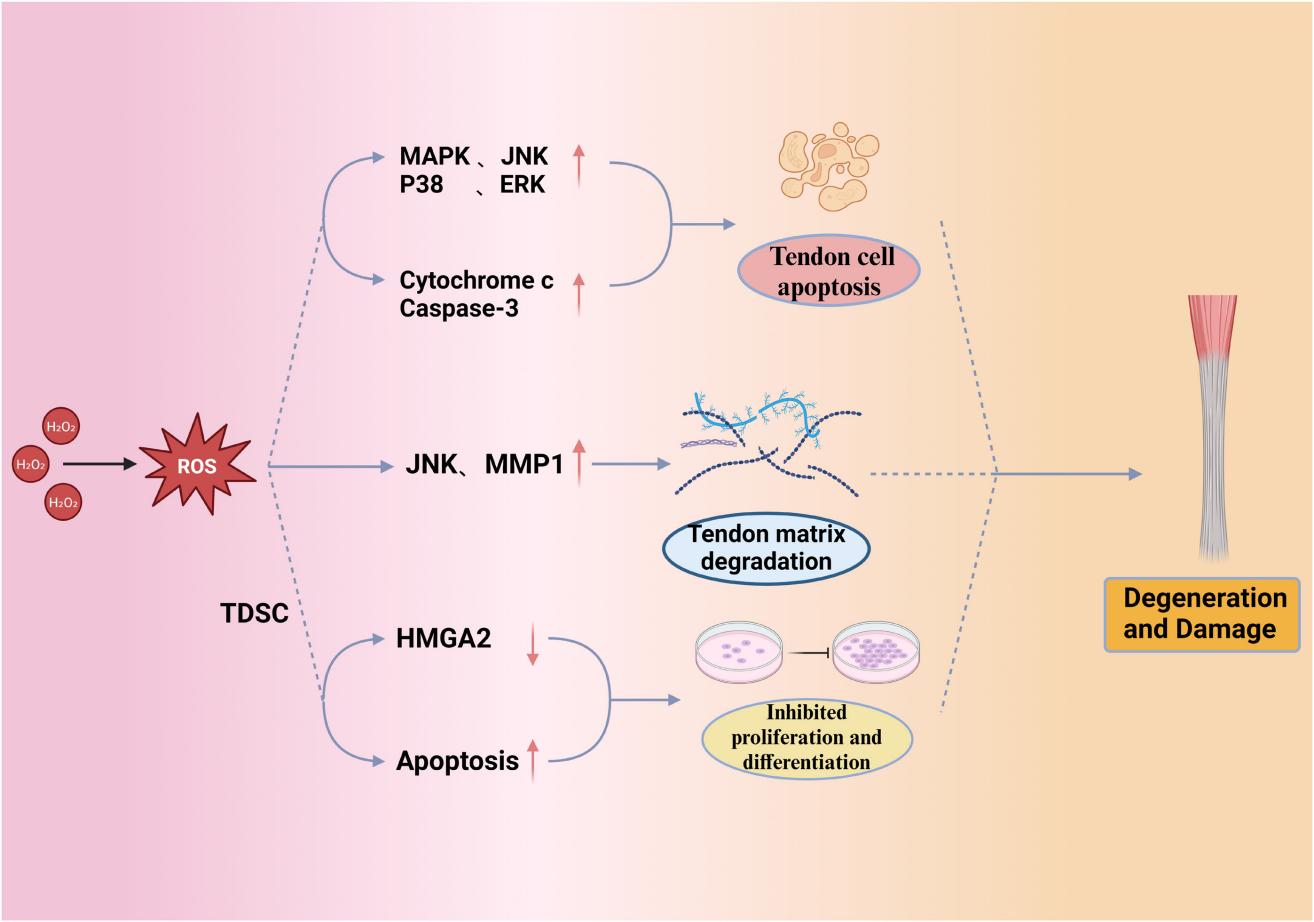
The relationship between ROS and tendinopathy. TDSCs, tendon‐derived stem cells; ROS, reactive oxygen species; MAPK, mitogen‐activated protein kinase; JNK, c‐Jun N‐terminal kinase; MMP1, matrix metallopeptidase 1.

In an early study by Wang et al.,^47^ they found that the antioxidative enzyme peroxiredoxin5 was upregulated in degenerating human tendons. Further research revealed that increased expression of peroxiredoxin5 could prevent apoptotic and functional loss of human tendon cells during oxidative stress.^45^ Yuan et al.^48^ employed proteomic approaches to study the pathogenesis of tendinopathy, finding significant differences in the levels of S100A11, PLIN4, and HYOU1 between the patient and control groups. Proteomic analysis suggested that these proteins are involved in oxidative stress and chronic inflammation. Yoshida et al.^49^ used superoxide anion fluorescent probes to measure superoxide levels in rotator cuff tissues and found higher levels in damaged tissues compared to normal ones. Similarly, Yazar et al.^50^ observed that serum total oxidative stress levels in patients with degenerative rotator cuff tears were higher than in healthy individuals. Morikawa et al.^51^ utilized superoxide dismutase (SOD) deficient mice to examine the effects of oxidative stress on rotator cuff degeneration, revealing a decrease in type I collagen and localized mechanical property degradation. Uehara et al.^52^ induced rotator cuff degeneration in rats and observed decreased levels of SOD and increased oxidative stress compared to controls. These clinical and animal studies suggest that oxidative stress may be a significant factor in rotator cuff degeneration and injury. Additionally, oxidative stress might also be related to re‐tearing post‐rotator cuff repair. Itoigawa et al.^53^ explored the correlation between oxidative stress and SOD levels in rotator cuff tissues with re‐tearing post‐surgery, finding elevated levels of both in the re‐tear group compared to the healing group (Figure 1).

### 2.1 Mechanisms of tendon degeneration and damage due to oxidative stress

1. Oxidative Stress Induces Tendon Cell Apoptosis: ROS have been identified as activators of the mitogen‐activated protein kinase (MAPK) signalling pathway, including JNK, P38, and ERK pathways, which promote tendon cell apoptosis. A study by Yuan et al. demonstrated that H_2_O_2_‐induced oxidative stress in human tendon cells leads to an increase in cytochrome c and caspase‐3 protein expression.^54^ This increase in H_2_O_2_ level causes cytochrome c release into the cytoplasm and elevates caspase‐3 activity, caspase‐3 plays a crucial role in the process of cellular apoptosis, which culminates in tendon cell apoptosis.

2. Oxidative Stress and Tendon Matrix Degradation: Research by Wang et al.^55^ revealed that oxidative stress induced by H_2_O_2_ increases the activity and mRNA expression of c‐Jun N‐terminal kinase (JNK) and matrix metallopeptidase 1 (MMP1) in human tendon cells. MMP1 can degrade collagen in vivo. This suggests a vital role of oxidative stress in the degradation of the tendon matrix.

3. Oxidative Stress Impairs Tendon‐Derived Stem Cells (TDSCs): Sun et al.^56^ induced oxidative stress in TDSCs using H_2_O_2_ and observed a reduction in HMGA2 protein expression, crucial for maintaining stem cell pluripotency. This affects stem cell proliferation and multidirectional differentiation. Similarly, Lee et al.^57^ found that oxidative stress caused by H_2_O_2_ promotes TDSC apoptosis, inhibiting cell survival and migration.

## 3 THE RELATIONSHIP BETWEEN OXIDATIVE STRESS AND OSTEOPOROSIS

**FIGURE 2:**
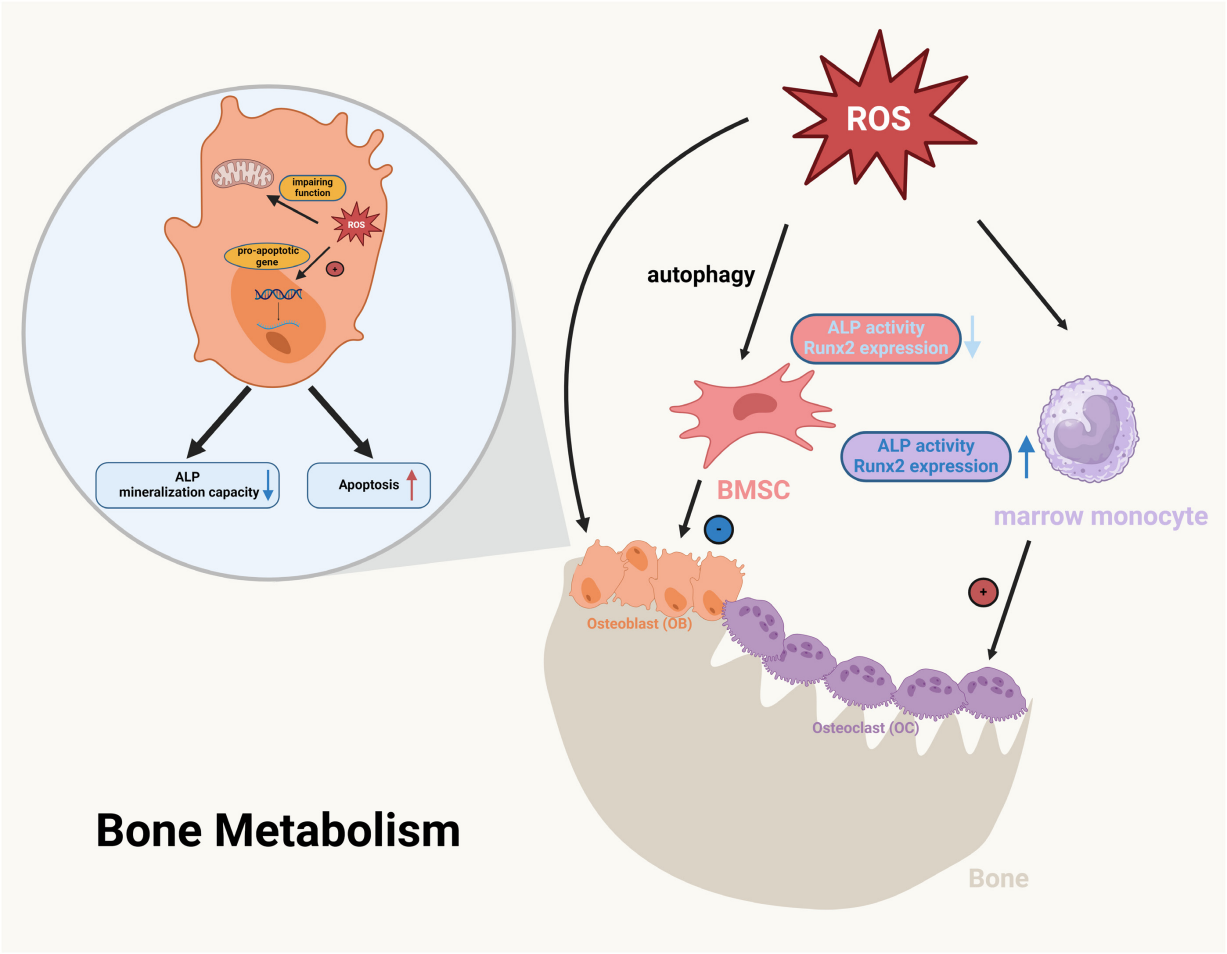
The Relationship between oxidative stress and osteoporosis.

Many clinical studies have confirmed that oxidative stress can promote osteoporosis. Cervellati et al.^58^ found a negative correlation between serum H_2_O_2_ levels and overall bone density in postmenopausal women, with further analysis revealing a connection between serum H_2_O_2_ levels and bone resorption markers. In research by Azizieh et al.,^59^ postmenopausal women were divided into groups based on bone density levels. They found significantly lower hydrogen peroxide enzyme and SOD2 levels in the group with abnormal bone density. A study by Yilmaz et al.^60^ involving 49 postmenopausal women showed that those with osteoporosis had lower serum total antioxidant levels and higher total peroxide levels compared to the non‐osteoporotic group. Jiang et al.^61^ studied 200 elderly osteoporotic individuals and 120 healthy controls, finding lower SOD levels and higher malondialdehyde levels in the osteoporotic group, correlating with bone density (Figure 2).

### 3.1 Mechanisms of oxidative stress in bone metabolism

1. Impact on Bone Marrow‐Derived Mesenchymal Stem Cells (BMSCs): Studies indicate that oxidative stress inhibits their proliferation and differentiation and promotes apoptosis. Geissler et al.^62^ discovered that BMSCs cultured long‐term in vitro exhibited reduced antioxidative abilities and increased ROS levels, resulting in a loss of osteogenic differentiation potential. Yang et al.^63^ showed that H_2_O_2_‐induced stress hindered osteogenic differentiation of BMSCs through the autophagy pathway, affecting Wnt/β‐catenin signalling. Similarly, Chen et al.^64^ observed reduced ALP activity and Runx2 expression in BMSCs under H_2_O_2_‐induced stress. Su et al.^65^ reported higher oxidative stress levels and reduced osteogenic differentiation in BMSCs from aged mice, which were improved with epigenetic therapies enhancing antioxidative levels.

2. Impairment of Osteoblast Function: Li et al.^66^ found that elevated ROS levels activate the JNK signalling pathway in osteoblasts, increasing the transcription of pro‐apoptotic genes, thereby promoting apoptosis and inhibiting bone formation. Tian et al.^67^ and Gan et al.^68^ demonstrated that oxidative stress impairs mitochondrial function in osteoblasts, reducing ALP expression, activity, and mineralisation capacity. Enhancing mitochondrial function and reducing ROS production can enhance osteoblast functionality.

3. Promotion of Osteoclast Formation and Differentiation: Studies show that ROS can directly or indirectly stimulate osteoclast differentiation, augmenting their activity and numbers, thereby enhancing bone resorption. Baek et al.^69^ co‐cultured H_2_O_2_ with human bone marrow monocytes, resulting in increased TRAP expression and upregulated M‐CSF and RANKL expression, promoting osteoclast differentiation and activity. Wan et al.^2^ reported consistent findings.

In summary, oxidative stress plays a crucial role in the pathogenesis of osteoporosis and tendinopathy by promoting apoptosis of tendon cells, tendon matrix degradation, and impairing tendon stem cell function. It also leads to metabolic imbalances in bones by inhibiting osteogenesis mediated by BMSCs and osteoblasts, whilst enhancing bone resorption mediated by osteoclasts.

## 4 THE POTENTIAL THERAPEUTIC VALUE OF ANTIOXIDANTS IN THE CO‐MORBIDITY TREATMENT OF OSTEOPOROSIS AND TENDINOPATHY

**FIGURE 3:**
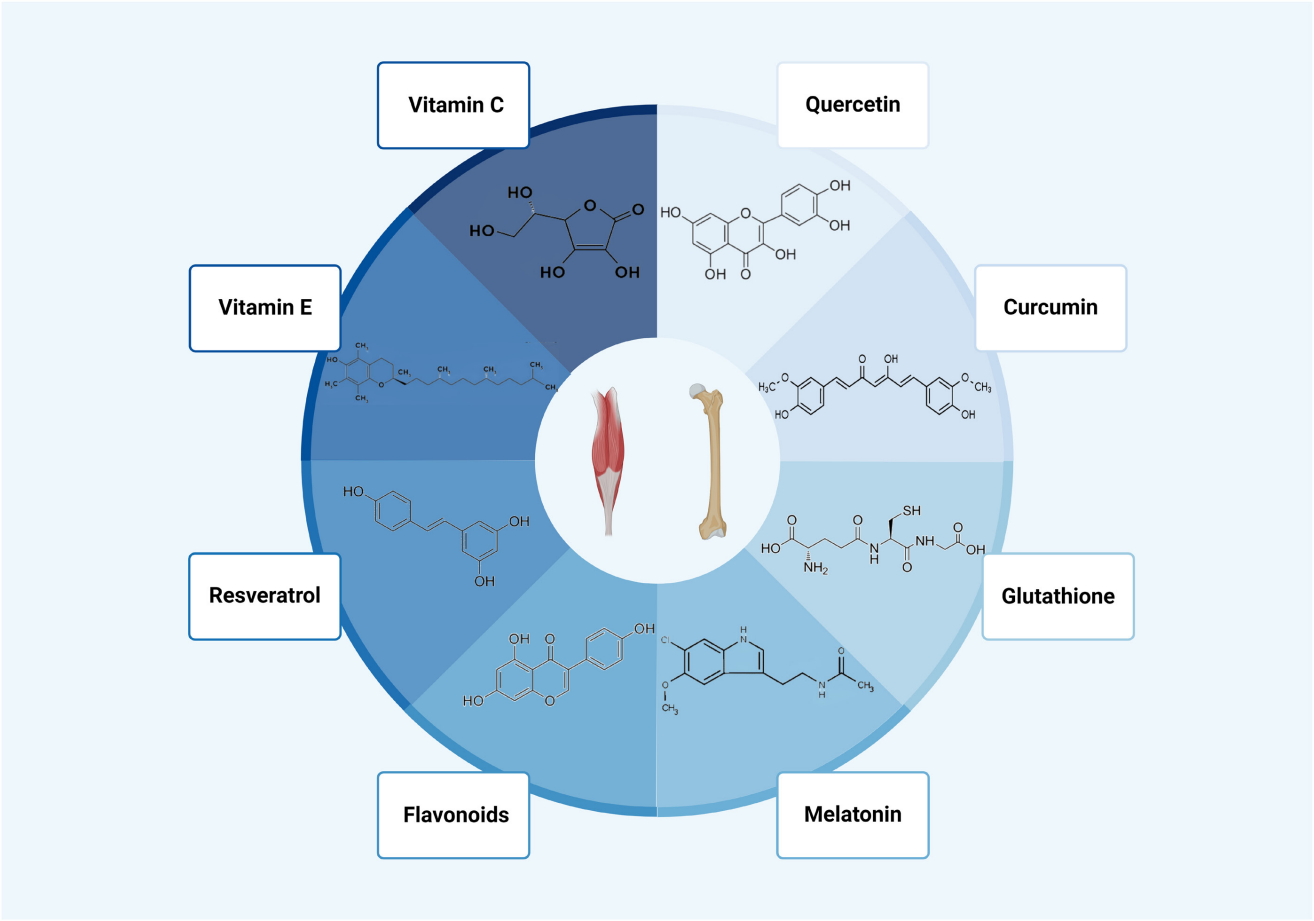
Potential therapeutic modalities for osteoporosis and tendinopathy: comorbidity mechanisms targeting oxidative stress.

Oxidative stress significantly influences the pathophysiological mechanisms of osteoporosis and tendinopathy co‐morbidities, highlighting antioxidant therapy as a potential treatment approach.^9^, ^19^ We have thoroughly explored how oxidative stress contributes to osteoporosis and tendon diseases. Next, let's delve into how antioxidants function in the treatment of these diseases and their impact on human health. Agents such as Vitamin C, melatonin, resveratrol, flavonoids and others are being explored for their potential effectiveness in treating these co‐morbid conditions (Figure 3).

### 4.1 Vitamin C

Known as L‐ascorbic acid, Vitamin C is a water‐soluble vitamin and functions as a potent antioxidant by neutralising oxygen free radicals. Research highlights its importance in the treatment of tendinopathy and osteoporosis. Morikawa et al.^70^ used SOD‐knockout mice to simulate rotator cuff degeneration and found that Vitamin C therapy effectively reduced oxidative stress in rotator cuff tissues, enhancing their biomechanical properties. Martel et al.^71^ in a clinical trial with 98 patients undergoing rotator cuff repair observed lower non‐healing rates in the Vitamin C group, only 11% of patients didn't heal, which was much lower than the 27% in the control group, indicating the potential for post‐surgical tendon healing. Mangano et al.^72^ reported correlations between plasma Vitamin C levels and bone density in postmenopausal women. Ruiz‐Ramos et al.^73^ observed that Vitamin C supplementation enhanced antioxidative capacity and bone density in the hip and spine. Li et al.^74^ demonstrated that Vitamin C intake in male adolescents could result in increased bone mass.

### 4.2 Melatonin

Melatonin, produced mainly by the pineal gland, possesses antioxidant, anti‐ageing, and immunomodulatory properties. Kocadal et al.^13^ assessed the impact of melatonin on supraspinatus tendinopathy, finding a reduction in total serum oxidative status and oxidative stress inhibition. Song et al.^75^ confirmed the efficacy of melatonin‐loaded electrospun membranes in tendon repair, improving biomechanical properties at the interface between tendon and bone. Yao et al.^76^ discovered that melatonin‐loaded scaffolds facilitated tendon healing by activating the Nrf2/HO‐1 pathway and reducing ROS and macrophage infiltration. Melatonin also plays a role in reducing oxidative stress and inflammation‐induced bone formation reduction whilst promoting bone resorption, presenting a novel target for osteoporosis treatment.^23^, ^77^ Lee et al.^78^ found that melatonin relieved the inhibition of BMSC osteogenic differentiation via the AMPK pathway. Chen et al.^79^ observed reduced oxidative stress levels in melatonin‐treated BMSCs from osteoporotic rats, promoting osteogenic differentiation and improving bone microarchitecture.

### 4.3 Resveratrol

This polyphenolic compound derived from plants, acts as a natural antioxidant. Moon et al.^80^ reported that resveratrol promoted osteogenic differentiation in human periosteal‐derived BMSCs by enhancing mitochondrial function. Zhou et al.^81^ confirmed resveratrol's role in inhibiting ROS production via the AMPK pathway, reducing BMSC senescence and promoting osteogenic differentiation. Clinical studies have shown that resveratrol is effective in slowing down the loss of lumbar and femoral neck bone mass and reducing hip fracture risks.^82^ Poulsen et al.^83^ found that resveratrol alleviated dexamethasone‐induced tendon cell ageing by activating Sirtuin‐1.

### 4.4 Flavonoids

These polyphenolic compounds with potent antioxidant properties promote BMSC bone formation via the Wnt and BMP2 signalling pathways and inhibit osteoclast differentiation and function, thereby playing a role in reducing bone loss.^20^ Bin et al.^46^ demonstrated that flavonoids inhibited H_2_O_2_‐induced apoptosis in rotator cuff tendinous cells by suppressing intracellular ROS production and modulating the JNK and ERK pathways. Song et al.^84^ reported that flavonoids attenuated oxidative damage in tendon‐derived stem cells induced by H_2_O_2_ by upregulating the Nrf‐2 pathway. Kim et al.^85^ indicated that flavonoids inhibited oxidative stress in rotator cuff fibroblasts caused by local anaesthetics.

### 4.5 Curcumin

Derived from the rhizomes of the Zingiberaceae and Araceae families, curcumin possesses anti‐inflammatory and antioxidant properties. Jiang et al.^86^ discovered that curcumin reduced oxidative stress in injured tendons, improving tendon healing quality. Chen et al.^87^ showed that curcumin‐loaded hydrogels exhibited anti‐inflammatory and antioxidant properties, promoting healing in rotator cuff tendon. Dai et al.^88^ discovered that curcumin improved mitochondrial function in osteoblasts, reducing apoptosis due to oxidative stress. Li^89^ observed that curcumin enhanced the GSK3β‐Nrf2 pathway inhibited by oxidative stress, thereby reducing ROS production in osteoblasts and promoting their formation and vitality. Xin et al.^90^ reported that curcumin suppressed microgravity‐induced ROS formation, enhancing osteoblast differentiation whilst inhibiting osteoclast differentiation.

### 4.6 Glutathione

This tripeptide is a major low‐molecular‐weight thiol in mammals, involved in detoxifying reactions against oxidants and electrophilic compounds. Yuan et al.^91^ found that glutathione played a crucial role in the development of osteoporosis in OVX mice. Probiotics affecting glutathione metabolism ameliorated osteoporosis induced by OVX in mice by regulating glutathione synthesis and reducing mitochondrial ROS production. Han et al.^92^ demonstrated that glutathione inhibited ROS, thereby attenuating osteoclast formation and preventing bone loss in osteoporotic mouse models. Glutathione inducers protect tendon cells from the impact of oxidative stress.^9^

### 4.7 Quercetin

Quercetin, a plant‐derived flavonoid compound, possesses antioxidant, anti‐apoptotic, and anti‐inflammatory properties. It enhances AMPK phosphorylation and upregulates SIRT1 expression, thus promoting the proliferation and osteogenic differentiation of mesenchymal stem cells.^93^ Xiao et al.^94^ found that quercetin activated the Nrf2/HO‐1 pathway, reducing apoptosis and ROS production to mitigate bone loss. Studies have shown that quercetin inhibits RANKL‐mediated osteoclast formation and osteoblast apoptosis.^95^ Yoshikawa et al.^96^ observed that quercetin reduced NOX expression in rat Achilles tendons, exerting antioxidant and anti‐inflammatory effects. Benjamin et al.^97^ demonstrated that quercetin downregulated the activation of MMP, ICAM‐1, and STAT3, protecting tendon injuries from oxidative stress, inflammation, apoptosis, and autophagy. Liang et al.^98^ found that quercetin improves tendon injuries by inhibiting oxidative stress.

### 4.8 Vitamin E

Vitamin E, known for its antioxidant and anti‐inflammatory properties, has been studied in various clinical settings. A trial showed that postmenopausal women supplementing with Vitamin E could slow the increase of bone resorption markers, mitigating bone loss.^74^ A cross‐sectional study found a correlation between low Vitamin E levels and osteoporosis in early postmenopausal women,^99^ women with low vitamin E levels experience decreased bone density and serum calcium concentration. Vakili et al.^100^ reported that Vitamin E improved OVX‐induced osteoporosis by reducing the expression of LC3, beclin1, and caspase3 whilst increasing bcl2 expression. When administered locally, water‐soluble analogs of Vitamin E reduced adhesions caused by tendon injuries.^101^

Additionally, antioxidants are widely used in treating various diseases.^102^, ^103^, ^104^, ^105^ In cardiovascular diseases, they reduce low‐density lipoprotein oxidation and prevent atherosclerosis formation.^106^ In cancer treatment, antioxidants inhibit the growth and proliferation of cancer cells.^107^, ^108^, ^109^ For certain arthritis forms, antioxidants mitigate inflammatory responses and alleviate pain.^110^ In neurodegenerative diseases, they protect neuronal cells from oxidative damage.^111^

## 5 SUMMARY AND PERSPECTIVE

Upon thoroughly reviewing the current scientific literature, the simultaneous prevalence of osteoporosis and tendinopathy in clinical settings is identified as a notable phenomenon, characterized by a significant degree of interrelation. A key shared characteristic between these two disorders is the significant role of oxidative stress as a shared etiological factor. Various antioxidants, ranging from Vitamin C and melatonin to resveratrol and anthocyanins, present promising potential as prospective treatments for these co‐occurring diseases. Additionally, traditional Chinese medicine formulations,^112^, ^113^ known for their antioxidative attributes, have shown emerging effectiveness in alleviating both osteoporosis and tendinopathy, as evidenced by existing studies.^114^, ^115^

However, incorporating antioxidants into the joint medical management of osteoporosis and tendinopathy presents several unresolved challenges. Whilst certain antioxidants have shown positive effects in treating either osteoporosis or tendinopathy individually, the collective evidence supporting their effectiveness in a comorbid context is unfortunately limited, both in human clinical trials and preclinical animal studies.^116^, ^117^ Furthermore, the complex and multifaceted nature of the pathophysiology underlying both osteoporosis and tendinopathy makes oxidative stress just one of many factors. This complexity requires more methodologically rigorous and comprehensive research to determine whether antioxidants should be used as stand‐alone treatments or as part of a broader, synergistic therapeutic approach. Recent advancements have also highlighted the potential role of artificial intelligence in expanding research in this area.^118^, ^119^

In summary, the significant role of oxidative stress in the co‐occurrence of osteoporosis and tendinopathy underscores its importance as a potential therapeutic target. Interventions targeting oxidative stress could pave the way for innovative treatment approaches to manage these interrelated conditions, highlighting the need for increased academic focus and empirical investigation.

## AUTHOR CONTRIBUTIONS

**Xianting Xia:** Funding acquisition (equal); writing – original draft (equal); writing – review and editing (equal). **Zhengyuan Fang:** Investigation (equal); resources (equal); writing – original draft (equal); writing – review and editing (equal). **Yinhua Qian:** Writing – original draft (equal); writing – review and editing (equal). **Yu Zhou:** Writing – review and editing (equal). **Haoqiang Huang:** Methodology (equal); writing – original draft (equal). **Feng Xu:** Data curation (equal); project administration (equal); writing – original draft (equal); writing – review and editing (equal). **Zhiwen Luo:** Conceptualization (equal); methodology (equal); resources (equal); writing – review and editing (equal). **Qing Wang:** Conceptualization (equal); methodology (equal); writing – original draft (equal).

## FUNDING INFORMATION

This work was supported Suzhou Clinical Key Disease Diagnosis and Treatment Technology Special Project (LCZX202127); Kunshan High‐level Medical Talent Program Project (Kunshan Health [2019] No. 6); Kunshan Chinese Medicine Science and Technology Development Special Project (KZYY2202); and Kunshan Hospital of Traditional Chinese Medicine Golden Apricot Superior Talent Project (03rczc25).

## CONFLICT OF INTEREST STATEMENT

The authors declare that the research was conducted in the absence of any commercial or financial relationships that could be construed as a potential conflict of interest.

## Data Availability

Data sharing is not applicable to this article as no new data were created or analyzed in this study.
